# Manganese Suppresses the Haploinsufficiency of Heterozygous *trpy1Δ/TRPY1* *Saccharomyces cerevisiae* Cells and Stimulates the TRPY1-Dependent Release of Vacuolar Ca^2+^ under H_2_O_2_ Stress

**DOI:** 10.3390/cells8020079

**Published:** 2019-01-22

**Authors:** Lavinia L. Ruta, Ioana Nicolau, Claudia V. Popa, Ileana C. Farcasanu

**Affiliations:** Department of Organic Chemistry, Biochemistry and Catalysis, Faculty of Chemistry, University of Bucharest, Sos. Panduri 90-92, 050663 Bucharest, Romania; lavinia.ruta@chimie.unibuc.ro (L.L.R.), ioana.nicolau@chimie.unibuc.ro (I.N.), valentina.popa@chimie.unibuc.ro (C.V.P.)

**Keywords:** TRP channel, TRPY1, *Saccharomyces cerevisiae*, calcium, manganese, oxidative stress

## Abstract

Transient potential receptor (TRP) channels are conserved cation channels found in most eukaryotes, known to sense a variety of chemical, thermal or mechanical stimuli. The *Saccharomyces cerevisiae* TRPY1 is a TRP channel with vacuolar localization involved in the cellular response to hyperosmotic shock and oxidative stress. In this study, we found that *S. cerevisiae* diploid cells with heterozygous deletion in *TRPY1* gene are haploinsufficient when grown in synthetic media deficient in essential metal ions and that this growth defect is alleviated by non-toxic Mn^2+^ surplus. Using cells expressing the Ca^2+^-sensitive photoprotein aequorin we found that Mn^2+^ augmented the Ca^2+^ flux into the cytosol under oxidative stress, but not under hyperosmotic shock, a trait that was absent in the diploid cells with homozygous deletion of *TRPY1* gene. TRPY1 activation under oxidative stress was diminished in cells devoid of Smf1 (the Mn^2+^-high-affinity plasma membrane transporter) but it was clearly augmented in cells lacking Pmr1 (the endoplasmic reticulum (ER)/Golgi located ATPase responsible for Mn^2+^ detoxification via excretory pathway). Taken together, these observations lead to the conclusion that increased levels of intracytosolic Mn^2+^ activate TRPY1 in the response to oxidative stress.

## 1. Introduction

Living cells are continuously exposed to various changes that threaten the dynamic equilibrium associated with the steady state of homeostatic balance. Such changes—often induced by stress agents—need to be sensed and signaled by cell components which belong to intricate networks responsible for homeostatic regulation. Calcium is a secondary messenger used by all eukaryotes—animal, plants, microorganisms—to connect various stimuli or stresses to their corresponding cellular responders. The budding yeast *Saccharomyces cerevisiae* has been constantly used as a model eukaryote to study the calcium-dependent response to various types of external stresses, which include salt [1], hypotonic [2,3], hypertonic [1,4,5], salicylate [6], alkaline [7], cold [8], ethanol [9,10], drugs [11] antifungals [12,13,14,15,16], electric [17] oxidative [18,19,20] or heavy metal [8,20,21,22] insults. The *S. cerevisiae* cells respond to such stresses by a sudden increase in cytosolic Ca^2+^—denoted henceforth [Ca^2+^]_cyt_—following the stimulus-dependent opening of Ca^2+^ channels situated in the plasma membrane and/or in internal compartments. Abrupt increase in [Ca^2+^]_cyt_ represents a versatile and universally used mechanism which triggers either cell survival/adaptation or cell death [23]. In *S. cerevisiae* the stress-dependent rise in [Ca^2+^]_cyt_ can be a consequence of Ca^2+^ influx via the Cch1/Mid1 channel on the plasma membrane [1,2] release of vacuolar Ca^2+^ into the cytosol through the vacuole-located Ca^2+^ channel TRPY1 [4,24], or both [19,20]. After delivering the message, the normal very low level of [Ca^2+^]_cyt_ is restored through the action of Ca^2+^ pumps and exchangers [25]. Thus, the Ca^2+^-ATPase Pmc1 [26] and a vacuolar Ca^2+^/H^+^ exchanger Vcx1 [27,28] independently transport [Ca^2+^]_cyt_ into the vacuole, while Pmr1, the secretory Ca^2+^-ATPase, pumps [Ca^2+^]_cyt_ into endoplasmic reticulum (ER) and Golgi along with Ca^2+^ extrusion from the cell [29,30].

In *S. cerevisiae*, TRPY1 is almost exclusively localized at the vacuolar membrane [4], playing an important role in adaptation to environmental stresses [4,19,20,21]. Initially named Yvc1, TRPY1 is encoded by *TRPY1* gene (systematic gene name, *YOR087W*) and it is the only member of the TRP (Transient Receptor Potential) superfamily of cationic channels expressed in *S. cerevisiae* [31]. TRP channels are conserved cation channels found in most eukaryotes, known to sense chemical, thermal, or mechanical stimuli in animals [32]. In yeast, TRPY1 is the main channel responsible for of [Ca^2+^]_cyt_ elevation under hyperosmotic shock [4,31], when calcium accrues predominantly from vacuolar stores [4]. This behavior can be explained by the mechano-sensitive traits of TRPY1: under hypertonic conditions water evacuates passively from the cytoplasm and then from the vacuole causing deformation of the vacuolar membrane and consequently the opening of the TRPY1 channel, with the release of vacuolar Ca^2+^ [5,33]. In contrast, under alkaline stress, the elevated [Ca^2+^]_cyt_ has its origin exclusively from the cell’s exterior, with the Cch1/Mid1 channel solely responsible for the majority of Ca^2+^ entry, and with no contribution of vacuolar Ca^2+^ [7]. In between these two situations, oxidative stress triggers [Ca^2+^]_cyt_ waves which pool both external and vacuolar Ca^2+^ [19]. TRPY1 is necessary for attaining a maximum level of [Ca^2+^]_cyt_ under oxidative stress and TRPY1 depends on [Ca^2+^]_cyt_ elevation for maximal gating, in a process known as Ca^2+^-induced Ca^2+^ release [34].

*TRPY1* gene is not essential for survival and the knockout mutant cells *trpy1Δ* have no clear growth defects under various stresses. Rather, it was shown that *trpy1Δ* cells are slightly more resistant to the oxidative stress imposed by exogenous hydrogen peroxide or tert-butylhydroperoxide [19] and Cu^2+^ [20] but also less fit under high Cd^2+^ [21] or tunicamycin-induced ER-stress in Ca^2+^-depleted medium [31]. In contrast, cells overexpressing the *TRPY1* gene are hypersensitive to surplus Ca^2+^ [4] or oxidative stress [19]. Also, it was revealed in a wide-scale survey that heterozygous *trpy1Δ/TRPY1* diploid cells are less fit under nutrient limiting conditions than the wild-type *TRPY1*/*TRPY1* ([35], Supplementary material). Haploinsufficiency occurs when the heterozygous mutation of a gene in a diploid organism results in a reduction of the corresponding gene product which can be correlated with negative alterations of the wild-type phenotype. In this study, we performed a chemical screen and found that non-toxic concentrations of Mn^2+^ alleviated the *trpy1Δ/TRPY1* haploinsufficiency observed by us in minimal growth medium containing half of the recommended amount of essential metal ions, probably by stimulating the TRPY1-mediated Ca^2+^ release into the cytosol. 

## 2. Materials and Methods

### 2.1. Yeast Strains and Growth Media

The *S. cerevisiae* diploid strains used in this study were isogenic with the “wild-type” (WT) parental strain BY4743 (*MAT*a/α; *his3Δ1*/*his3Δ1*; *leu2Δ0*/*leu2Δ0*; *met15Δ0*/*MET15*; *LYS2*/*lys2Δ0*; *ura3Δ0*/*ura3Δ0*), a S288C-based yeast strain [36]. The strains harbored either heterozygous (BY4732, *orf::kanMX4*/*ORF)* or homozygous (BY4732, *orf::kanMX4*/*orf::kanMX4)* knockout mutations of individual gene open reading frames (ORF). The heterozygous knockout mutants are referred to in the text as *orfΔ*/*ORF* and were *cch1Δ*/*CCH1*, *mid1Δ*/*MID1*, *pmc1Δ*/*PMC1*, *pmr1Δ*/*PMR1*, *vcx1Δ*/*VCX1*, *trpy1Δ*/*TRPY1*. The homozygous knockout mutants are referred to in the text as *orfΔ*/*orfΔ* and were *trpy1Δ*/*trpy1Δ*, *smf1Δ*/*smf1Δ*, and *pmr1Δ*/*pmr1Δ*. The strains were obtained from EUROSCARF (European *S. cerevisiae* Archive for Functional Analysis, www.euroscarf.de) and were propagated, grown, and maintained in YPD medium (1% yeast extract, 2% polypeptone, 2% glucose) or SD (0.17% yeast nitrogen base without amino acids, 0.5% (NH_4_)_2_SO_4_, 2% glucose, supplemented with the necessary amino acids) [37]. The strains transformed with the plasmids harboring apo-aequorin cDNA [38] were selected and maintained on SD lacking uracil (SD-Ura). Minimal defined media (MM) were prepared adding individual components as described [37] using ultrapure reagents (Merck, Darmstadt, Germany) and contained 1 mM Ca^2+^, 0.25 µM Cu^2+^, 2 µM Mn^2+^, 2 µM Fe^3+^ and 2 µM Zn^2+^. Low-metal minimal defined medium (LMeMM) had 0.5 mM Ca^2+^, 0.1 µM Cu^2+^, 1 µM Mn^2+^, 1 µM Fe^3+^ and 1 µM Zn^2+^, corresponding roughly to half of the amount of essential metals recommended [37]. The concentrations of metals in MM and LMeMM were confirmed by inductively coupled plasma with mass spectrometry (ICP-MS, Perkin-Elmer ELAN DRC-e, Concord, ON, Canada) against Multielement ICP Calibration Standard 3, matrix 5% HNO_3_ (Perkin-Elmer Pure Plus, Shelton, CT, USA). All synthetic media had their pH adjusted to 6.5. For solid media, 2% agar was used. For growth improvement, all the synthetic media were supplemented with an extra 20 mg/L leucine [39]. All chemicals, including media reagents were from Merck (Darmstadt, Germany),

### 2.2. Plasmid and Yeast Transformation

For heterologous expression of aequorin, yeast cells were transformed with the multicopy *URA3*-based plasmid pYX212-*cytAEQ* harboring the apo-aequorin cDNA under the control of the strong *TPI* (triosephosphate isomerase) yeast promoter [40]. Plasmid pYX212-*cytAEQ* was a generous gift from Martegani and Tisi (University of Milano-Bicocca, Milan, Italy). Yeast transformation [41] was performed using S.c. EasyComp™ Transformation Kit (Invitrogen, Carlsbad, CA, USA) following manufacturer’s indications. 

### 2.3. Yeast Cell Growth Assay

#### 2.3.1. Growth in Liquid Media

Unless otherwise specified, cells were incubated at 30 °C under agitation (200 rpm). Yeast strains were pre-grown overnight in MM then diluted (1/20) in fresh MM and grown for 2 h. Cells were harvested by centrifugation, washed with ice-cold water, and resuspended in liquid LMeMM at density which corresponded to optical density measured at 600 nm (OD_600_) = 0.05. Strain growth in liquid LMeMM was monitored in time by measuring OD_600_ recorded in a plate reader equipped with thermostat and shaker (Varioskan, Thermo Fisher Scientific, Vantaa, Finland). Relative growth was expressed as the ratio between OD_600_ recorded at time *t* and OD_600_ recorded at time 0. For screening of chemicals against *trpy1Δ*/*TRPY1* haploinsufficiency (HIP), cells shifted to LMeMM (OD_600_ = 0.05) were incubated for 2 h before chemicals were added from concentrated sterile stocks. Cell growth (%) was determined 24 h from chemical addition and calculated relatively to growth (OD_600_) of WT strain, no added chemicals. Chemicals used were CuCl_2_, FeCl_2_, MnCl_2_, ZnCl_2_, ascorbate, ethylene glycol-bis(2-aminoethylether)-*N*,*N*,*N′*,*N′*-tetraacetic acid (EGTA), GdCl_3_, glutathione (GSH), indole and were all of high-grade purity. 

#### 2.3.2. Growth on Solid Media

For dilution plate assay, exponentially growing cells were 10-fold serially diluted in a 48-well microtiter plate and stamped on agar plates using a pin replicator (approximately 4 μL/spot). Plates were photographed after incubation at 30 °C for 3 days. 

### 2.4. TRPY1 Gene Expression by Quantitative Reverse Transcriptase-Polymerase Chain Reaction (qRT-PCR)

Wild-type cells BY4743 (*TRPY1/TRPY1*), heterozygous (*trpy1Δ/TRPY1*), and homozygous (*trpy1Δ/trpy1Δ*) diploid cells from overnight pre-cultures were inoculated at OD_600_ = 0.1 in MM or LMeMM and grown to OD_600_ = 0.5 before Mn^2+^ was added to final concentration 10 µM, then incubated for 2 additional hours before being harvested for total ribonucleic acid (RNA) isolation. Total RNA was isolated using the RiboPure™ RNA Purification Kit for yeast (Ambion™, Thermo Fischer Scientific, Vilnius, Lithuania) following the manufacturer’s instructions. Approximately 500 ng of total RNA was transcribed into cDNA using GoScript™ Reverse Transcription System (Promega, Madison, WI, USA). Finally, a total of 10 ng cDNA was used for each qRT-PCR done with the GoTaq^®^ qPCR Master Mix (Promega). Each reaction was performed in triplicate using MyiQ Single-Color Real-Time PCR Detection System (BioRad, Hercules, CA, USA). Expression of *TRPY1* mRNA was normalized to the relative expression of *ACT1* in each sample. The qRT-PCR cycling conditions were 95 °C for 1 min, and 40 cycles of 95 °C for 10 s, 59 °C for 10 s, 72 °C for 12 s. The primers used for amplification of cDNA were: TRPY1-F: 5′-AGATTCTCAG GGTTACGTTA, TRPY1-R: 5′-CAATATGGAATACCACTCAC; ACT1-F: 5′-GGTTGCTGCTTTGGTTATTG, ACT1-R: 5′-CAATTGGGTAACGTAAAGTC.

### 2.5. Assay of Cell Mn^2+^

Measurements of cell total manganese content were done on cells grown in LMeMM medium to an OD_600_ of 1.0. Cells were harvested in triplicate samples, washed two times in ice-cold 10 mM 2-(*N*-morpholino) ethanesulfonic acid (MES)-Tris buffer (pH 6.0). Cells were finally suspended in deionized water (OD_600_ = 10) and used for manganese and cell protein assay. Manganese analysis was done by ICP-MS after digestion of cells with 65% ultrapure HNO_3_ (Merck, Darmstadt, Germany). The metal cellular content was normalized to total cellular proteins, as described [42]. Total cellular manganese was expressed as nanomoles of metal per mg cell protein.

### 2.6. Detection of [Ca^2+^]_cyt_ by Aequorin Bioluminescence Assay

Cells transformed with pYX212-*cytAEQ* were maintained on SD-Ura selective medium and prepared for Ca^2+^ dependent luminescence detection as described [43] with slight modifications. Overnight pre-cultures of cells expressing apo-aequorin were washed and suspended (OD_600_ = 0.5) in LMeMM-Ura then incubated to late exponential phase (OD_600_ = 1, 4–6 h). For pre-incubation with Mn^2+^, MnCl_2_ was added to the desired concentration and cells were grown for an additional 2 h. Cells were harvested by centrifugation and resuspened (to OD_600_ = 10) in LMeMM-Ura in which the corresponding Mn^2+^ concentration was maintained. To reconstitute functional aequorin, native coelenterazine was added to the cell suspension (from a methanol stock, 20 µM final concentration) and the cells were incubated for 2 h at 30 °C in the dark. The excess coelenterazine was removed by centrifugation. The cells (approximately 10^7^ cells/determination) were finally resuspended in LMeMM-Ura with corresponding Mn^2+^ concentration and transferred to the luminometer tube. A cellular luminescence baseline was determined for each strain by approximately one minute of recordings at 1/s intervals. After ensuring a stable signal, chemicals tested were injected from sterile stocks to give the final concentrations indicated, and the Ca^2+^-dependent light emission was monitored in a single tube luminometer (Turner Biosystems, 20^n^/20, Sunnyvale, CA, USA). The light emission was measured at 1 s intervals and expressed as relative luminescence units (RLU). To ensure that the total reconstituted aequorin was not limiting in our assay, at the end of each experiment aequorin activity was checked by lysing cells with 1% Triton X-100 with 5 mM CaCl_2_ and only the cells with considerable residual luminescence were considered. Relative luminescence emission was normalized to an aequorin content giving a total light emission of 10^6^ RLUs in 10 min after lysing cells with 1% Triton X-100. The relative luminescence maximum (RLM) was the average of the RLUs flanking the maximum value minus the average luminescence baseline recorded before cells were exposed to the stimulus (10 values on each side), all normalized as described above.

### 2.7. Statistics

All experiments were repeated, independently, in three biological replicates at least. For each individual experiment, values were expressed as the mean ± standard error of the mean (SEM). For aequorin luminescence determinations, traces represent the mean ± SEM from three independent transformants. The numerical data were examined by Student *t* test or by analysis of variance with multiple comparisons (ANOVA) using the statistical software Prism version 6.05 for Windows (GraphPad Software, La Jolla, CA, USA). The differences were considered to be significant when *p* < 0.05. One sample *t* test was used for the statistical analysis of each strain/condition compared with a strain/condition considered as reference. Asterisks indicate the level of significance: * *p* < 0.05, ** *p* < 0.01, and *** *p* < 0.001.

## 3. Results

### 3.1. Haploinsufficiency of Yeast Strain Heterozyous for TRPY1 Is Alleviated by Mn^2+^

**Figure 1:**
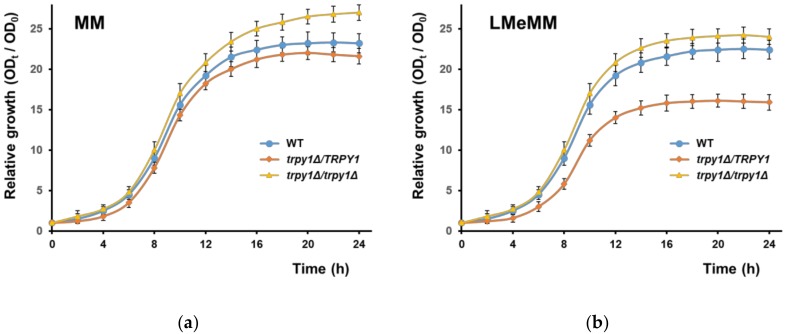
Growth of heterozygous *trpy1Δ*/*TRPY1*. Isogenic diploid strains WT (BY4743, *TRPY1*/*TRPY1*), *trpy1Δ*/*TRPY1* and *trpy1Δ*/*trpy1Δ* were shifted at time 0 to (**a**) minimal medium, MM or (**b**) minimum medium with low metal content, LMeMM, as described in Materials and Methods section. Growth was determined spectrophotometrically at 600 nm as the ratio between OD_600_ at time *t* and OD_600_ at time 0 for each individual strain.

To highlight new aspects related to TRPY1 function in yeast cells, the main target of our study was to identify conditions which interfere with TRPY1 activity. In this direction, haploinsufficiency is a genetic trait which can be very useful in the attempts to identify small molecules which influence the behavior of functional proteins [44]. A genome-wide survey had already pinpointed the heterozygous *trpy1Δ*/*TRPY1* as possibly less fit under nutrient limiting conditions ([35], Supplementary material). We noticed that the growth of the heterozygous *trpy1Δ*/*TRPY1* diploid mutant was not significantly different from the growth of the wild-type diploid when the two strains were incubated in YPD, SD (data not shown) or MM medium (Figure 1a), but *trpy1Δ*/*TRPY1* cells exhibited somehow slower growth (*p* < 0.001) in minimal synthetic medium LMeMM which had approximately half of the amount of essential metals recommended [37] (Figure 1b). 

**Figure 2:**
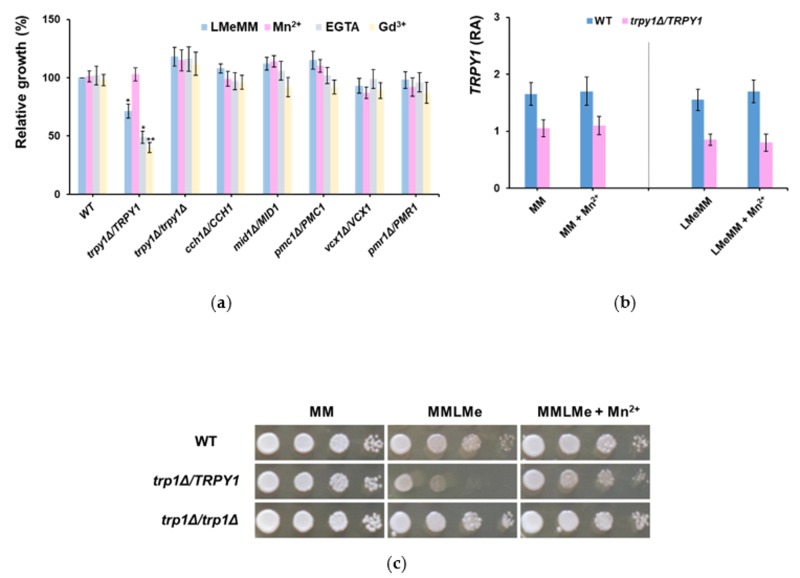
Haploinsufficiency of heterozygous *trpy1Δ*/*TRPY1*. (**a**) Mn^2+^ alleviates *trpy1Δ*/*TRPY1* haploinsufficiency in LMeMM. Diploid strains were shifted to LMeMM (final OD_600_ = 0.05) and grown for 2 h before MnCl_2_ (10 µM), EGTA (0.5 mM) or GdCl_3_ (50 µM) were added from concentrated stocks. Cell growth was recorded spectrophotometrically 24 h after the addition of the chemicals and normalized (%) to the growth of WT in the absence of chemicals. One sample *t* test compared WT in the absence of chemicals. * *p* < 0.05; ** *p* < 0.01. (**b**) Relative abundance (RA) of *TRPY1* mRNA in WT (*TRPY1*/*TRPY1*) and heterozygous *trpy1Δ*/*TRPY1*. Analysis of transcript abundance was done by qRT-PCR as described in Materials and Methods section. Expression of *TRPY1* mRNA was normalized to the relative expression of *ACT1* in each sample. (**c**) Heterozygous *trpy1Δ*/*TRPY1* exhibits haploinsufficiency in LMeMM, but not in normal MM. Cells in log phase (OD_600_ ~ 0.5) were 10-fold serially diluted (left to right) in a 48-well microtiter plate and stamped on the agar plates using a pin replicator (approximately 4 µL/spot). Plates were photographed after 3 days’ incubation at 30 °C.

The haploinsufficiency in LMeMM was noted only for *TRPY1*; no similar phenotype was recorded for heterozygous strains with mutations in the genes which encode the other transporters known to participate in regulating [Ca^2+^]_cyt_, e.g., *CCH1*, *MID1*, *PMC1* or *VCX1* (Figure 2a, dark blue bars). 

To identify compounds which potentially interact with TRPY1 activity we screened for conditions which may alleviate or augment the haploinsufficient phenotype observed. The tested substances are presented in Table 1. These substances were added to the LMeMM at the point where the heterozygous *trpy1Δ*/*TRPY1* diploid cells were in the early log phase of growth (OD_600_ = 0.1) and the effect on growth was determined 24 h after chemical addition. We tested the effect of adding physiological concentrations of the metals initially depleted in LMeMM (i.e., Ca^2+^, Cu^2+^, Fe^3+^, Mn^2+^, Zn^2+^) but also of glutathione and indole, which had been reported to interact with TRPY1 [45,46]. As glutathione is a universal intracellular antioxidant, we also tested an exogenous antioxidant, i.e., ascorbate. EGTA was chosen as a chelator of Ca^2+^ in the growth medium, while Gd^3+^ was tested as a blocker of the Ca^2+^ channels. The results showing the effect of the tested compounds on *trpy1Δ*/*TRPY1* haploinsufficiency in LMeMM are included in Supplementary Files, Figure S1. 

Out of the compounds tested, only Mn^2+^ alleviated the *trpy1Δ*/*TRPY1* haploinsufficiency observed in LMeMM. In contrast, EGTA and Gd^2+^ augmented the LMeMM-associated growth defect (Figure 2a). The level of *TRPY1* gene expression was lower in *trpy1Δ*/*TRPY1* compared with wild-type, but this level was not significantly influenced by surplus Mn^2+^ (Figure 2b), suggesting that Mn^2+^ acts—directly or indirectly—by activating the TRPY1 channel. The *trpy1Δ*/*TRPY1* haploinsufficiency was also noted on solid LMeMM. In contrast, the *trpy1Δ*/*trpy1Δ* growth was not affected (Figure 2c).

### 3.2. Mn^2+^ Potentiates the Increase of [Ca^2+^]_cyt_ under Oxidative Stress in Strain trpy1Δ/TRPY1 

**Figure 3:**
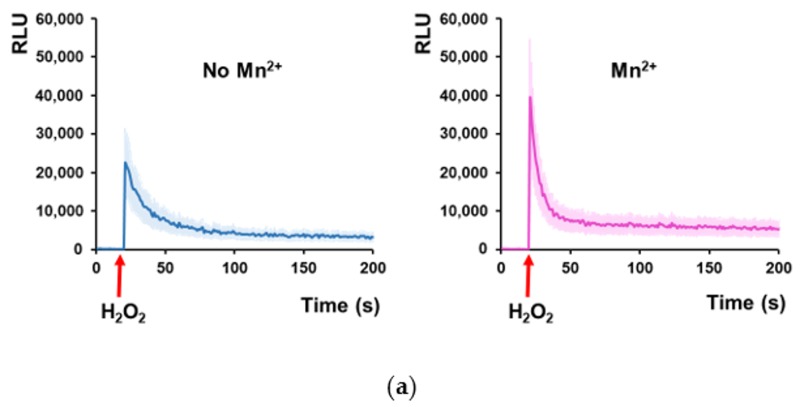

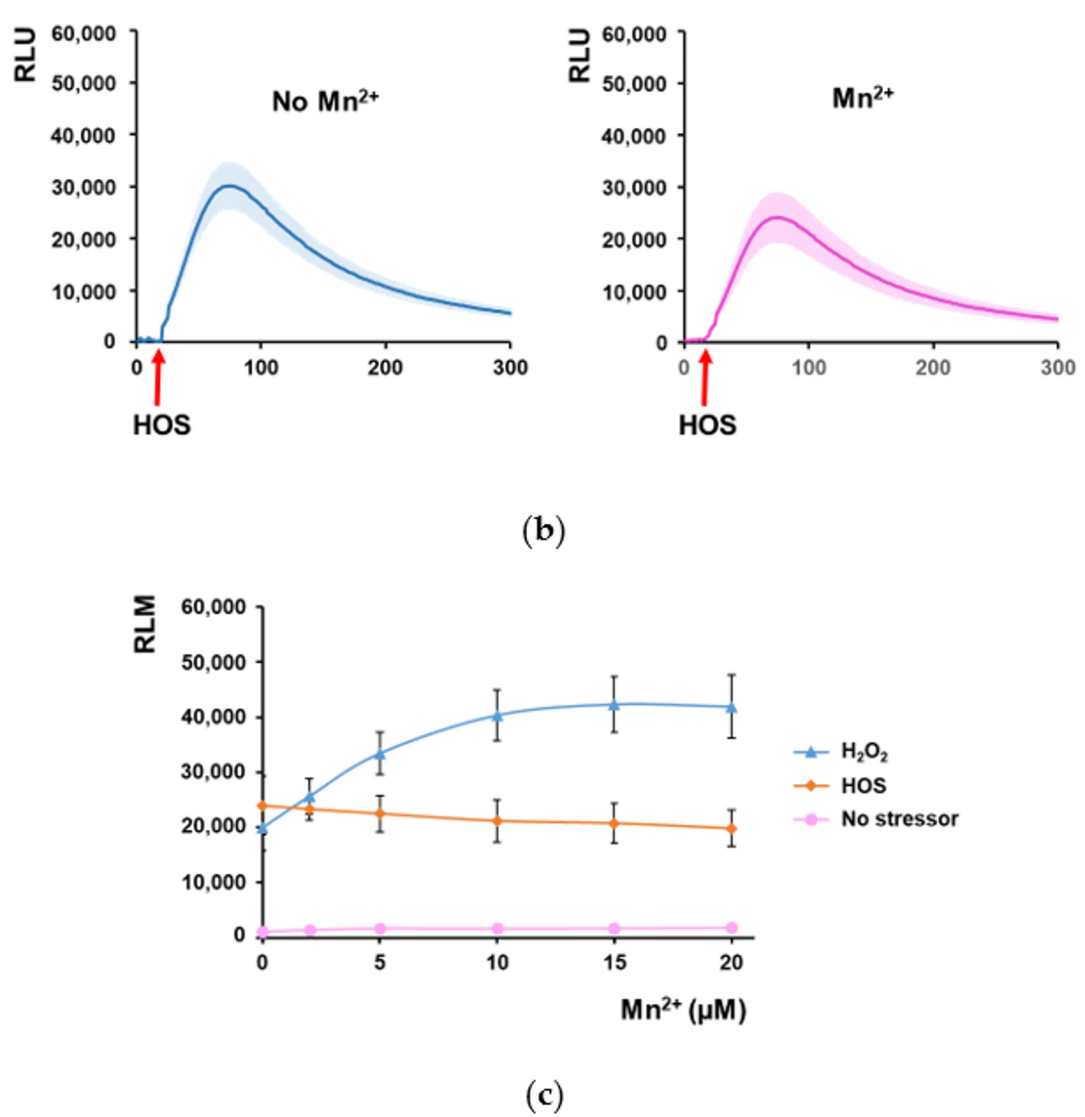
In *trpy1Δ*/*TRPY1* cells, Mn^2+^ pre-incubation stimulates the increase of [Ca^2+^]_cyt_ under H_2_O_2_ stress but not under hyperosmotic shock. Heterozygous *trpy1Δ*/*TRPY1* cells expressing reconstituted aequorin were pre-grown in LMeMM-Ura without or with 10 µM surplus Mn^2+^ before being exposed to (**a**) oxidative stress (5 mM H_2_O_2_) or (**b**) hyperosmotic stress (HOS, 0.8 M NaCl). [Ca^2+^]_cyt_-dependent aequorin luminescence was recorded on samples of approximately 10^7^ cells. The arrow indicates the time when the stressor was added. The luminescence traces represent the mean ± SEM from 3 independent transformants. RLU, relative luminescence units. (**c**) Effect of pre-incubation with various concentrations of Mn^2+^ on the maximum intensity of the Ca^2+^-dependent aequorin luminescence recorded under 5 mM H_2_O_2_, or 0.8 M NaCl (HOS). The relative maximum luminescence (RLM) was calculated as described in Materials and Methods. Bars represent the mean ± SEM from 3 independent transformants.

The observation that both EGTA (calcium chelator) and Gd^3+^ (inhibitor of Ca^2+^ transport across plasma membrane) augmented the LMeMM-related haploinsufficiency of the *trpy1Δ/TRPY1* strain prompted the idea that preventing Ca^2+^ entry into the cell is deleterious to *trpy1Δ/TRPY1*, while the observed opposite action of Mn^2+^ may be the result of Mn^2+^-related activation of the extant TRPY1 that would compensate the heterozygous loss of *TRPY1*. To check this possibility, we used cells expressing aequorin, a system suitable for detecting transient modifications in the [Ca^2+^]_cyt_ [38]. For this purpose, *trpy1Δ/TRPY1* cells were transformed with a plasmid harboring the cDNA of the luminescent Ca^2+^ reporter apo-aequorin under the control of a constitutive promoter, which afforded abundant transgenic protein within the cytosol [40]. The cells expressing apo-aequorin were pre-treated with the cofactor coelenterazine to reconstitute the functional aequorin, and then the cells were exposed to various stimuli directly in the luminometer tube. It was noted that while Mn^2+^ alone failed to induce any increase in the luminescence of the reconstituted aequorin (data not shown), cell pre-incubation with 10 µM Mn^2+^ significantly increased the [Ca^2+^]_cyt_ elevation induced by H_2_O_2_ exposure (Figure 3a). Remarkably, pre-incubation with Mn^2+^ did not augment the cell luminescence when aequorin-expressing *trpy1Δ/TRPY1* cells were exposed to hyperosmotic shock (HOS, Figure 3b,c). Surplus Mn^2+^ reached maximum stimulating activity on *trpy1Δ/TRPY1* cells exposed to H_2_O_2_ at 10 µM (Figure 3c), a non-toxic concentration to both WT and *trpy1* mutants.

### 3.3. Mn^2+^ Stimulates TRPY1 to Release Ca^2+^ into the Cytosol under H_2_O_2_ Stress

**Figure 4:**
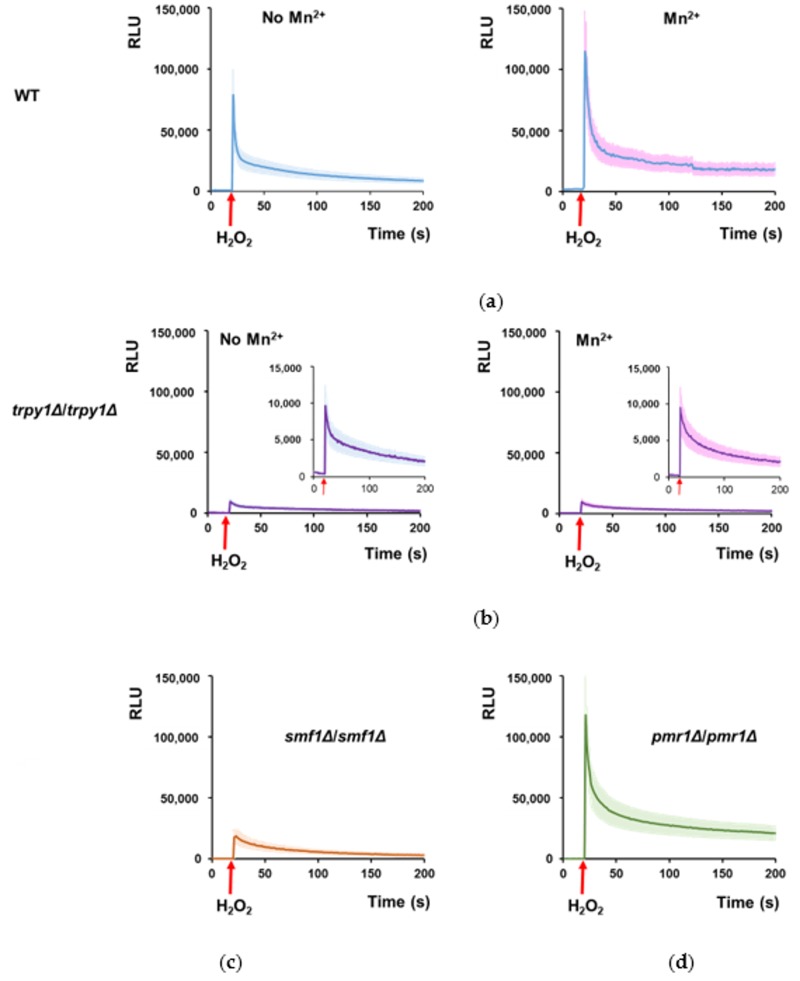
Variation of [Ca^2+^]_cyt_ in response to H_2_O_2_ exposure depends on Mn^2+^ cellular load. Diploid cells expressing reconstituted aequorin were pre-grown in LMeMM-Ura with or without 10 µM surplus Mn^2+^ before being exposed to oxidative stress (5 mM H_2_O_2_) as described in Materials and Methods. [Ca^2+^]_cyt_-dependent aequorin luminescence was recorded on samples of approximately 10^7^ cells. The arrow indicates the time when the stressor (H_2_O_2_) was added. The luminescence traces represent the mean ± SEM from 3 independent transformants. (**a**) WT (BY4743). (**b**) *trpy1Δ*/*trpy1Δ*; insets: same representation at lower scale. (**c**) *smf1Δ*/*smf1Δ*. (**d**) *pmr1Δ*/*pmr1Δ*. RLU, relative luminescence units.

Furthermore, we wondered whether Mn^2+^ influence on elevating [Ca^2+^]_cyt_ under H_2_O_2_ was the result of TRPY1 stimulation by Mn^2+^. To test this possibility, we determined the effect of Mn^2+^ on the Ca^2+^-mediated response to H_2_O_2_ of cells completely lacking TRPY1. It was noticed that in WT cells expressing reconstituted aequorin, the H_2_O_2_-induced Ca^2+^-dependent luminescence was significantly increased by cell pre-incubation with 10 µM Mn^2+^, indicating that in the case of WT cells too, Mn^2+^ potentiates the Ca^2+^-dependent response to oxidative stress (Figure 4a). In contrast, homozygous knockout mutant *trpy1Δ*/*trpy1Δ* exhibited much lower H_2_O_2_-luminescence (Figure 4b, left), which was not altered by pre-incubation with 10 µM Mn^2+^ (Figure 4b, right). This observation suggested that Mn^2+^ augments the H_2_O_2_-induced [Ca^2+^]_cyt_ elevation by activating TRPY1, a phenotype clearly absent in the *trpy1Δ*/*trpy1Δ* homozygous knockout mutant (Figure 4b). 

If Mn^2+^ were required for TRPY1 activation under oxidative stress, it would be expected that cells with low cytosolic Mn^2+^ would be less responsive in terms of increasing the [Ca^2+^]_cyt_ under oxidative stress. Mn^2+^ is an essential metal which is carried into the yeast cell by the divalent metal ion transporter Smf1, known to have high affinity for Mn^2+^ [47]. It was noted indeed that the homozygous knockout mutant *smf1Δ*/*smf1Δ* expressing reconstituted aequorin exhibited a significantly lower luminescence trace when exposed to H_2_O_2_ than WT (Figure 4c). In this line of evidence, the *pmr1Δ*/*pmr1Δ* cells expressing reconstituted aequorin responded strongly to H_2_O_2_ (in media not supplemented with Mn^2+^) with a luminescence curve (Figure 4d) which was not significantly different from that obtained from WT cells preincubated with 10 µM Mn^2+^ (Figure 4a, right). *PMR1* encodes the major Golgi/ER membrane P-type ATPase ion pump responsible for transporting Ca^2+^ and Mn^2+^ into the Golgi apparatus [48] providing a major route for cellular detoxification of Mn^2+^ via the secretory pathway vesicles [49]. It was shown that cells knockout for *PMR1* gene have the intracellular Mn^2+^ levels considerably higher than the WT cells [50], a fact that may account for the stronger response of *pmr1Δ*/*pmr1Δ* cells (Figure 4d) compared to WT (Figure 4a, left). In this line of evidence, we found that *pmr1Δ*/*pmr1Δ* cells had significantly (*p* < 0.05) more cellular Mn^2+^ than the WT, while *smf1Δ*/*smf1Δ* cell had significantly (*p* < 0.05) less cellular Mn^2+^ than the WT (Table 2).

**Figure 5:**
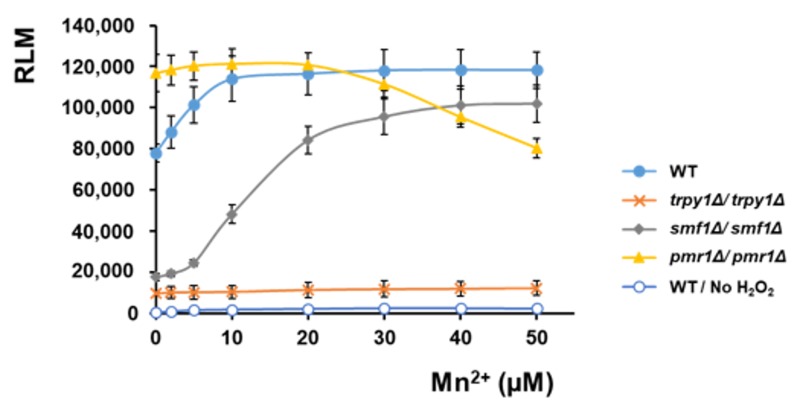
Effect of Mn^2+^ pre-incubation on the maximum intensity of the Ca^2+^-dependent aequorin luminescence recorded for various strains under H_2_O_2_ stress. The relative maximum luminescence (RLM) was calculated as described in Materials and Methods. Diploid cells expressing reconstituted aequorin were pre-grown in LMeMM-Ura with or without surplus Mn^2+^ before being exposed to oxidative stress (5 mM H_2_O_2_) directly in the luminometer tube. [Ca^2+^]_cyt_-dependent aequorin luminescence was recorded on samples of approximately 10^7^ cells. Bars represent the mean ± SEM from 3 independent transformants.

The influence of Mn^2+^ on the RLM recorded under oxidative stress for various strains which expressed reconstituted aequorin was also determined (Figure 5), revealing that Mn^2+^ significantly increased the RLM of WT cells exposed to H_2_O_2_. RLM determined for *trpy1Δ/trpy1Δ* was significantly low and was not augmented by Mn^2+^, indicating the necessity of functional TRPY1 for Mn^2+^ action. RLM for *smf1Δ*/*smf1Δ* cells expressing reconstituted aequorin was also low under H_2_O_2_ exposure, indicating that the lack of the high-affinity Mn^2+^ transporter is associated with cytosolic Mn^2+^ concentration (Table2) which is too low for an efficient activation of TRPY1. In fact, *smf1Δ*/*smf1Δ* attained responses similar to WT only at higher Mn^2+^ supplementation (Figure 5, grey line), when Mn^2+^ cell content was high enough (Table 2) for efficient TRPY1 activation. In contrast to *smf1Δ*/*smf1Δ* strain, *pmr1Δ*/*pmr1Δ* expressing reconstituted aequorin attained high RLM upon H_2_O_2_ exposure which was not significantly augmented by surplus Mn^2+^, suggesting that the intrinsic high level of cytosolic Mn^2+^ associated with *PMR1* knockout [50] is sufficient for attaining efficient activation of TRPY1 (Table2). Moreover, it was noted that when applying Mn^2+^ concentrations higher than 20 µM the maximum response of *pmr1Δ*/*pmr1Δ* to H_2_O_2_ started to decline (Figure 5, yellow line) probably due to the hypersensitivity of this strain to Mn^2+^ [50]. 

## 4. Discussion

TRPY1 of *S. cerevisiae* is a key component in releasing vacuolar Ca^2+^ into the cytosol for the Ca^2+^-dependent activation of mechanisms involved in the cell response to hyperosmotic [4] and oxidative stress [19]. Starting from the observation that Mn^2+^ alleviated the haploinsufficiency exhibited by the heterozygous *trpy1Δ*/*TRPY1* strain in synthetic media deficient in essential metals (LMeMM) we found that Mn^2+^ differentially stimulated TRPY1 to release Ca^2+^ from the vacuole under H_2_O_2_ exposure, but not under hyperosmotic shock. Mn^2+^ alone does not induce [Ca^2+^]_cyt_ elevation—neither under low (0.05–1 mM) nor under high (2–10 mM, lethal) surplus [21, unpublished observations]. The Mn^2+^ concentrations found to augment the H_2_O_2_-induced stimulation of TRPY1 were within the physiological limits (10–50 µM) and far below the concentration that would induce a hyperosmotic shock, explaining why the TRPY1 was not extra stimulated by Mn^2+^ under hyperosmotic stress (Figure 3b). It was shown that the release of vacuolar Ca^2+^ via TRPY1 can be stimulated by Ca^2+^ from outside the cell as well as that released from the vacuole by TRPY1 itself in a positive feedback, a process known as Ca^2+^-induced Ca^2+^ release [34,51]. In this regard, Mn^2+^ surplus could stimulate TRPY1 similarly to Ca^2+^. Mn^2+^ is an essential cation which strongly resembles Ca^2+^ not only in ionic radius but also in its affinity to oxygen-containing ligands, a trait which sometimes makes Mn^2+^ a good substitute of Ca^2+^ [52]; this would explain why other essential cations tested (Cu^2+^, Fe^3+^, Zn^2+^) failed to alleviate the haploinsufficiency showed by *trpy1Δ*/*TRPY1* strain. That *TRPY1* haploinsufficiency in LMeMM is rescued by Mn^2+^ can be explained in three ways: (1) the supplemental Mn^2+^ simply counteracts the deficiency of essential metals of the LMeMM, providing the necessary amount of cations (albeit surrogate in certain cases) that support cell fitness; (2) Mn^2+^ stimulates TRPY1 activity by increasing the Ca^2+^ release to the cytososl, and consequently by stimulating other components involved in maintaining the cell fitness; (3) Mn^2+^ generates reactive oxygen species (ROS) by a Fenton-like reaction augmenting the oxidative stress and indirectly stimulating TRPY1. The fact that surplus Mn^2+^ augments the TRPY1-related increase in [Ca^2+^]_cyt_ under oxidative stress clearly correlates with the Mn^2+^ cytosolic level, as the strain lacking the high-affinity plasma membrane Mn^2+^ transporter Smf1 exhibited only a modest increase in [Ca^2+^]_cyt_ under H_2_O_2_, when compared with WT (Figure 4c). In this line of evidence, it was shown that a haploid *smf1Δ* was sensitive to H_2_O_2_ [20] probably by not attaining the optimum TRPY1 activation for adaptation to oxidative stress. On the other hand, it had been shown that deletion of *PMR1*—which leads to increased cytosolic Mn^2+^—suppresses the sensitivity of superoxide dismutase (SOD) mutants to superoxide-generating drugs due to the Mn^2+^ capacity to scavenge superoxide ions [50]. In the light of our findings, it is also possible that the high cytosolic Mn^2+^ in cells devoid of Pmr1 rescue the SOD mutants from ROS attack not due to the scavenger traits of Mn^2+^, but through TRPY1 activation. Whether Mn^2+^ rescues the haploinsufficient *trpy1Δ*/*TRPY1* by neutralizing ROS or by directly stimulating TRPY1 are issues to be addressed in the future; nevertheless, the observation that well-known antioxidants such as ascorbate or glutathione did not rescue the *trpy1Δ*/*TRPY1* haploinsufficiency rather supports the latter hypothesis. An open question remains: why is the homozygous *trpy1Δ*/*trpy1Δ* apparently more fit than the heterozygous *trpy1Δ*/*TRPY1*. The calcium-mediated responses to environmental insults are diverse: depending on the intensity or duration of the [Ca^2+^]_cyt_ waves, the cell can be led towards adaptation, survival (sometimes with growth arrest or slow growth) or death [19,20,21,23]. The *trpy1Δ*/*trpy1Δ* cells are probably more fit because they are never “bothered” by periodic nuisance caused by Ca^2+^ release from the vacuole; on the other hand, *trpy1Δ*/*TRPY1* cells need to find the right balance in Ca^2+^ gating while depending on only one gene copy, and sometimes extra help—external Ca^2+^ carried by Cch1/Mid1, mechanical force [34,51] or even surplus Mn^2+^—may contribute to find the most suitable path to be followed. 

## Figures and Tables

**Table 1:** Substances screened for the capacity to alleviate *trpy1Δ*/*TRPY1* haploinsufficiency in LMeMM.

Substance Tested ^1^	Concentration Range	Effect on *trpy1Δ*/*TRPY1* Haploinsufficiency
CaCl_2_	2–10 mM	No
CuCl_2_	0.5–50 µM	No
FeCl_3_	1–50 µM	No
MnCl_2_	1–50 µM	Alleviation
ZnCl_2_	1–50 µM	No
EGTA	0.1–2 mM	Augmentation
GdCl_3_	0.1–1 mM	Augmentation
Ascorbate	1–10 mM	No
Glutathione ^2^	1–10 mM	No
Indole ^3^	1–10 mM	No

^1^ The quantitative results are presented in Supplementary Files, Figure S1. ^2^ Glutathione depletion leads to TRPY1 activation [45]. ^3^ Indole activates TRPY1 under hyperosmotic stress [46].

**Table 2:** Manganese content (pmoles/mg cell protein) of diploid yeast cells grown in LMeMM supplemented or not with Mn^2+^.

Strain	Surplus Mn^2+^		
	0	10 µM	50 µM
WT	0.12 ± 0.12	0.64 ± 0.2	0.92 ± 0.3
*trpy1Δ*/*TRPY1*	0.11 ± 0.14	0.72 ± 0.1	0.98 ± 0.2
*trpy1Δ*/*trpy1Δ*	0.12 ± 0.2	0.7 ± 0.2	0.84 ± 0.2
*smf1Δ*/*smf1Δ*	0.01 ± 0.014	0.1 ± 0.2	0.72 ± 0.2
*pmr1Δ*/*pmr1Δ*	0.7 ± 0.22	0.84 ± 0.2	8.4 ± 1.2

## Supplementary Materials

The following are available online. Figure S1: Effect of various substances on the haploinsufficiency of the heterozygous *trpy1Δ*/*TRPY1*.

## References

[1] Matsumoto T.K., Ellsmore A.J., Cessna S.G., Low P.S., Pardo J.M., Bressan R.A., Hasegawa P.M. (2002). An osmotically induced cytosolic Ca^2+^ transient activates calcineurin signaling to mediate ion homeostasis and salt tolerance of *Saccharomyces cerevisiae*. J. Biol. Chem..

[2] Batiza A.F., Schulz T., Masson P.H. (1996). Yeast respond to hypotonic shock with a calcium pulse. J. Biol. Chem..

[3] Rigamonti M., Groppi S., Belotti F., Ambrosini R., Filippi G., Martegani E., Tisi R. (2015). Hypotonic stress-induced calcium signaling in Saccharomyces cerevisiae involves TRP-like transporters on the endoplasmic reticulum membrane. Cell Calcium.

[4] Denis V., Cyert M.S. (2002). Internal Ca^2+^ release in yeast is triggered by hypertonic shock and mediated by a TRP channel homologue. J. Cell Biol..

[5] Loukin S., Zhou X., Kung C. (2008). Saimi, Y. A genome-wide survey suggests an osmoprotective role for vacuolar Ca^2+^ release in cell wall-compromised yeast. FASEB J..

[6] Mori I.C., Iida H., Tsuji F.I., Isobe M., Uozumi N., Muto S. (1998). Salicylic acid induces a cytosolic Ca^2+^ elevation in yeast. Biosci. Biotechnol. Biochem..

[7] Viladevall L., Serrano R., Ruiz A., Domenech G., Giraldo J., Barcelo A., Arino J. (2004). Characterization of the calcium-mediated response to alkaline stress in *Saccharomyces cerevisiae*. J. Biol. Chem..

[8] Peiter E., Fischer M., Sidaway K., Roberts S.K., Sanders D. (2005). The *Saccharomyces cerevisiae* Ca^2+^ channel Cch1pMid1p is essential for tolerance to cold stress and iron toxicity. FEBS Lett..

[9] Araki Y., Wu H., Kitagaki H., Akao T., Takagi H., Shimoi H. (2009). Ethanol stress stimulates the Ca^2+^-mediated calcineurin/Crz1 pathway in *Saccharomyces cerevisiae*. J. Biosci. Bioeng..

[10] Courchesne W.E., Vlasek C., Klukovich R., Coffee S. (2011). Ethanol induces calcium influx via the Cch1-Mid1 transporter in *Saccharomyces cerevisiae*. Arch. Microbiol..

[11] Gupta S.S., Ton V.K., Beaudry V., Rulli S., Cunningham K., Rao R. (2003). Antifungal activity of amiodarone is mediated by disruption of calcium homeostasis. J. Biol. Chem..

[12] Rao A., Zhang Y.Q., Muend S., Rao R. (2010). Mechanism of antifungal activity of terpenoid phenols resembles calcium stress and inhibition of the TOR pathway. Antimicrob. Agents Chemother..

[13] Hejchman E., Ostrowska K., Maciejewska D., Kossakowski J., Courchesne W.E. (2012). Synthesis and antifungal activity of derivatives of 2- and 3-benzofurancarboxylic acids. J. Pharmacol. Exp. Ther..

[14] Roberts S.K., McAinsh M., Widdicks L. (2012). Cch1p mediates Ca^2+^ influx to protect *Saccharomyces cerevisiae* against eugenol toxicity. PLoS ONE.

[15] Roberts S.K., McAinsh M., Cantopher H., Sandison S. (2014). Calcium dependence of eugenol tolerance and toxicity in *Saccharomyces cerevisiae*. PLoS ONE.

[16] Popa C.V., Lungu L., Cristache L.F., Ciuculescu C., Danet A.F., Farcasanu I.C. (2015). Heat shock, visible light or high calcium augment the cytotoxic effects of Ailanthus altissima (Swingle) leaf extracts against Saccharomyces cerevisiae cells. Nat. Prod. Res..

[17] Vilanova C., Hueso A., Palanca C., Marco G., Pitarch M., Otero E., Crespo J., Szablowski J., Rivera S., Domínguez-Escribà L. (2011). Aequorin-expressing yeast emits light under electric control. J. Biotechnol..

[18] Pinontoan R., Krystofova S., Kawano T., Mori I.C., Tsuji F.I., Iida H., Muto S. (2002). Phenylethylamine induces an increase in cytosolic Ca^2+^ in yeast. Biosci. Biotechnol. Biosci..

[19] Popa C.V., Dumitru I., Ruta L.L., Danet A.F., Farcasanu I.C. (2010). Exogenous oxidative stress induces Ca^2+^ release in the yeast *Saccharomyces cerevisiae*. FEBS J..

[20] Ruta L.L., Popa C.V., Nicolau I., Farcasanu I.C. (2016). Calcium signaling and copper toxicity in *Saccharomyces cerevisiae* cells. Environ. Sci. Pollut. Res. Int..

[21] Ruta L.L., Popa V.C., Nicolau I., Danet A.F., Iordache V., Neagoe A.D., Farcasanu I.C. (2014). Calcium signaling mediates the response to cadmium toxicity in *Saccharomyces cerevisiae* cells. FEBS Lett..

[22] Ene C.D., Ruta L.L., Nicolau I., Popa C.V., Iordache V., Neagoe A.D., Farcasanu I.C. (2015). Interaction between lanthanide ions and *Saccharomyces cerevisiae* cells. J. Biol. Inorg. Chem..

[23] Bootman M.D., Berridge M.J., Putney J.W., Llewelyn Roderick H. (2011). Calcium Signaling.

[24] Palmer C.P., Zhou X., Lin J., Loukin S.H., Kung C., Saimi Y. (2001). A TRP homolog in *Saccharomyces cerevisiae* forms an intracellular Ca^2+^-permeable channel in the yeast vacuolar membrane. Proc. Natl. Acad. Sci. USA.

[25] Cunningham K.W. (2011). Acidic calcium stores of *Saccharomyces cerevisiae*. Cell Calcium.

[26] Cunningham K.W., Fink G.R. (1994). Calcineurin-dependent growth control in *Saccharomyces cerevisiae* mutants lacking *PMC1*, a homolog of plasma membrane Ca^2+^ ATPases. J. Cell Biol..

[27] Cunningham K.W., Fink G.R. (1996). Calcineurin inhibits VCX1-dependent H^+^/Ca^2+^ exchange and induces Ca^2+^ ATPases in *Saccharomyces cerevisiae*. Mol. Cell Biol..

[28] Miseta A., Kellermayer R., Aiello D.P., Fu L., Bedwell D.M. (1999). The vacuolar Ca^2+^/H^+^ exchanger Vcx1p/Hum1p tightly controls cytosolic Ca^2+^ levels in *S. cerevisiae*. FEBS Lett..

[29] Sorin A., Rosas G., Rao R. (1997). PMR1, a Ca^2+^-ATPase in yeast Golgi, has properties distinct from sarco/endoplasmic reticulum and plasma membrane calcium pumps. J. Biol. Chem..

[30] Strayle J., Pozzan T., Rudolph H.K. (1999). Steady-state free Ca^2+^ in the yeast endoplasmic reticulum reaches only 10 mM and is mainly controlled by the secretory pathway pump Pmr1. EMBO J..

[31] Hamamoto S., Mori Y., Yabe I., Uozumi N. (2018). In vitro and in vivo characterization of modulation of the vacuolar cation channel TRPY1 from *Saccharomyces cerevisiae*. FEBS J..

[32] Clapham D.E. (2003). TRP channels as cellular sensors. Nature.

[33] Zhou X.L., Batiza A.F., Loukin S.H., Palmer C.P., Kung C., Saimi Y. (2003). The transient receptor potential channel on the yeast vacuole is mechanosensitive. Proc. Natl. Acad. Sci. USA.

[34] Chang Y., Schlenstedt G., Flockerzi V., Beck A. (2010). Properties of the intracellular transient receptor potential (TRP) channel in yeast, Yvc1. FEBS Lett..

[35] Pir P., Gutteridge A., Wu J., Rash B., Kell D.B., Zhang N., Oliver S.G. (2012). The genetic control of growth rate: A systems biology study in yeast. BMC Syst. Biol..

[36] Brachmann C.B., Davies A., Cost G.J., Caputo E., Li J., Hieter P., Boeke J.D. (1998). Designer deletion strains derived from *Saccharomyces* cerevisiae S288C: A useful set of strains and plasmids for PCR-mediated gene disruption and other applications. Yeast.

[37] Sherman F. (2002). Getting started with yeast. Methods Enzymol..

[38] Nakajima-Shimada J., Iida H., Tsuji F.I., Anraku Y. (1991). Monitoring of intracellular calcium in *Saccharomyces cerevisiae* with an apoaequorine cDNA expression system. Proc. Natl. Acad. Sci. USA.

[39] Cohen R., Engelberg D. (2007). Commonly used *Saccharomyces cerevisiae* strains (e.g. BY4741, W303) are growth sensitive on synthetic complete medium due to poor leucine uptake. FEMS Microbiol. Lett..

[40] Tisi R., Baldassa S., Belotti F., Martegani E. (2002). Phospholipase C is required for glucose-induced calcium influx in budding yeast. FEBS Lett..

[41] Dohmen R.J., Strasser A.W.M., Honer C.B., Hollenberg C.P. (1991). An efficient transformation procedure enabling long-term storage of competent cells of various yeast genera. Yeast.

[42] Ruta L.L., Kissen R., Nicolau I., Neagoe A.D., Petrescu A.J., Bones A.M., Farcasanu I.C. (2017). Heavy metal accumulation by *Saccharomyces cerevisiae* cells armed with metal binding hexapeptides targeted to the inner face of the plasma membrane. Appl. Microbiol. Biotechnol..

[43] Tisi R., Martegani E., Brandão R.L. (2015). Monitoring yeast intracellular Ca^2+^ levels using an in vivo bioluminescence assay. Cold Spring Harb. Protoc..

[44] Petrovic K., Pfeifer M., Parker C.N., Schuierer S., Tallarico J., Hoepfner D., Movva N.R., Scheel G., Helliwell S.B. (2017). Two low complexity ultra-high throughput methods to identify diverse chemically bioactive molecules using Saccharomyces cerevisiae. Microbiol. Res..

[45] Chandel A., Das K.K., Bachhawat A.K. (2016). Glutathione depletion activates the yeast vacuolar transient receptor potential channel, Yvc1p, by reversible glutathionylation of specific cysteines. Mol. Biol. Cell.

[46] John Haynes W., Zhou X.L., Su Z.W., Loukin S.H., Saimi Y., Kung C. (2008). Indole and other aromatic compounds activate the yeast TRPY1channel. FEBS Lett..

[47] Supek F., Supekova L., Nelson H., Nelson N. (1996). A yeast manganese transporter related to the macrophage protein involved in conferring resistance to mycobacteria. Proc. Natl. Acad. Sci. USA.

[48] Dürr G., Strayle J., Plemper R., Elbs S., Klee S.K., Catty P., Wolf D.H., Rudolph H.K. (1998). The medial-Golgi ion pump Pmr1 supplies the yeast secretory pathway with Ca^2+^ and Mn^2+^ required for glycosylation, sorting, and endoplasmic reticulum-associated protein degradation. Mol. Biol. Cell.

[49] Mandal D., Woolf T.B., Rao R. (2000). Manganese selectivity of Pmr1, the yeast secretory pathway ion pump, is defined by residue Gln783 in transmembrane segment 6. Residue Asp778 is essential for cation transport. J. Biol. Chem..

[50] Lapinskas P.J., Cunningham K.W., Liu X.F., Fink G.R., Culotta V.C. (1995). Mutations in *PMR1* suppress oxidative damage in yeast cells lacking superoxide dismutase. Mol. Cell. Biol..

[51] Su Z., Zhou X., Loukin S.H., Saimi Y., Kung C. (2009). Mechanical force and cytoplasmic Ca^2+^ activate yeast TRPY1 in parallel. J. Membr. Biol..

[52] Loukin S., Kung C. (1995). Manganese effectively supports yeast cell-cycle progression in place of calcium. J. Cell Biol..

