# Supplementary material for: Manganese Suppresses the Haploinsufficiency of Heterozygous trpy1Δ/TRPY1 Saccharomyces cerevisiae Cells and Stimulates the TRPY1-Dependent Release of Vacuolar Ca2+ under H2O2 Stress
Source: Cells. 2019 Jan 22;8(2):79. doi: 10.3390/cells8020079 (PMC6406398; doi:10.3390/cells8020079)
Supplement: Supplementary file 1 [file cells-08-00079-s001.pdf]

cells

MDPI

Manganese Suppresses the Haploinsufficiency of Heterozygous *trpy1Δ/TRPY1 Saccharomyces cerevisiae* Cells and Stimulates the TRPY1-Dependent Release of Vacuolar Ca^{2+} under H_2O_2 Stress

Lavinia L. Ruta, Ioana Nicolau, Claudia V. Popa and Ileana C. Farcasanu*

Department of Organic Chemistry, Biochemistry and Catalysis, Faculty of Chemistry, University of Bucharest, Sos. Panduri 90-92, 050663 Bucharest, Romania; lavinia.ruta@chimie.unibuc.ro (L.L.R.), ioana.nicolau@chimie.unibuc.ro (I.N.), valentina.popa@chimie.unibuc.ro (C.V.P.)

* Correspondence: ileana.farcasanu@chimie.unibuc.ro; Tel.: +40-721-067-169

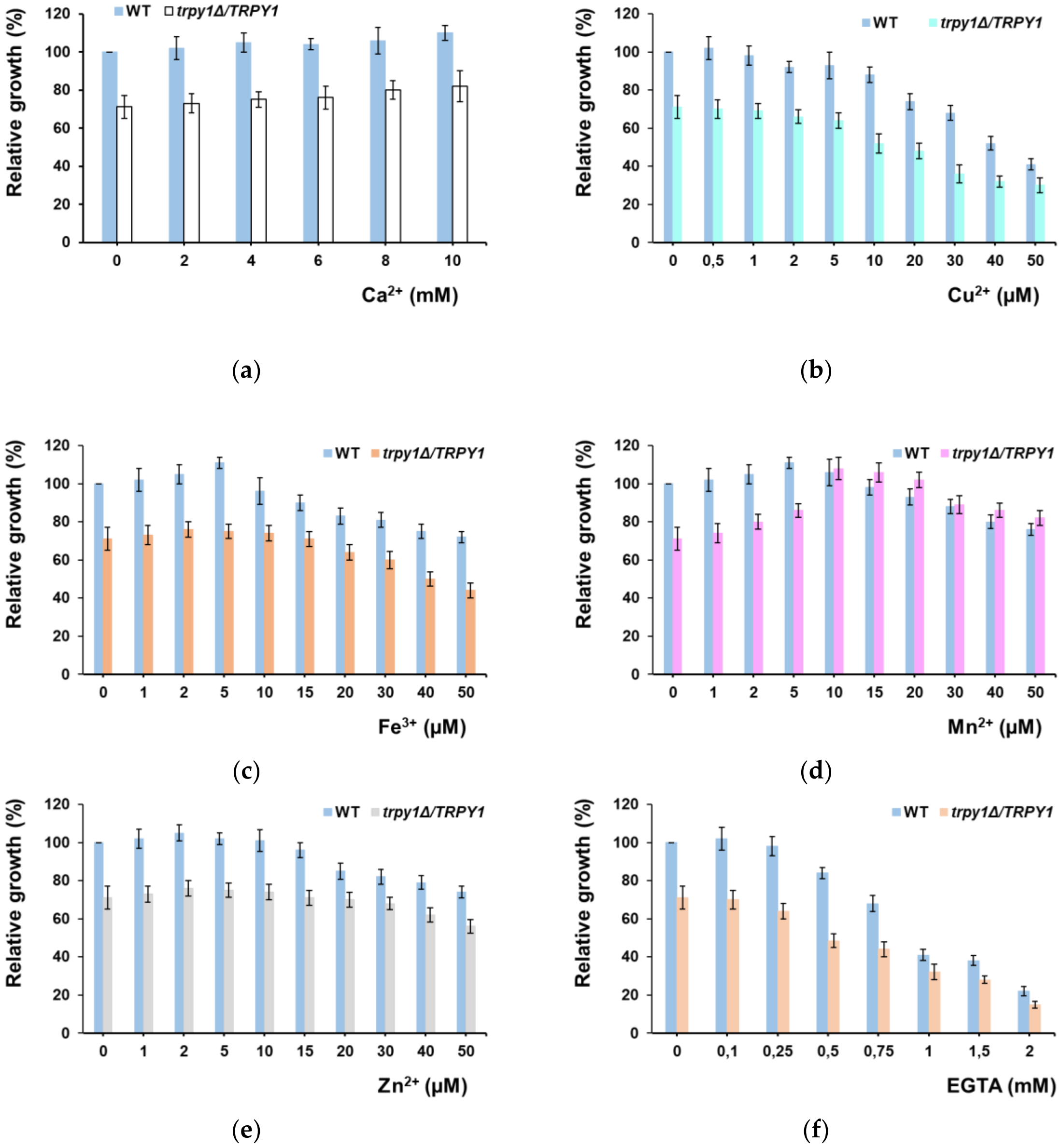

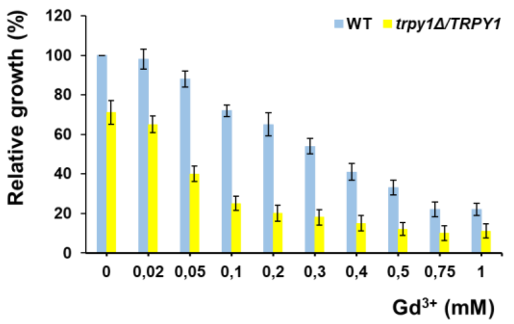

(**g**)

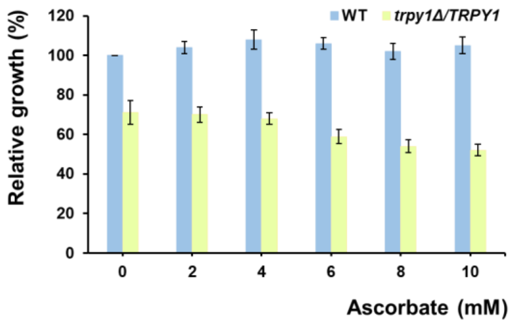

(**h**)

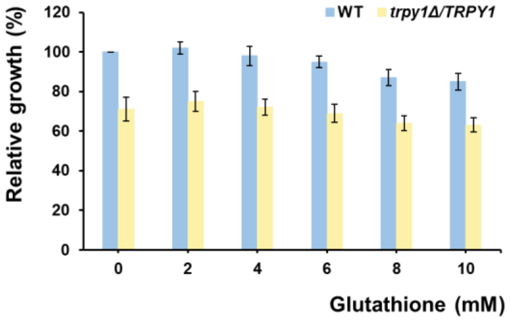

(**i**)

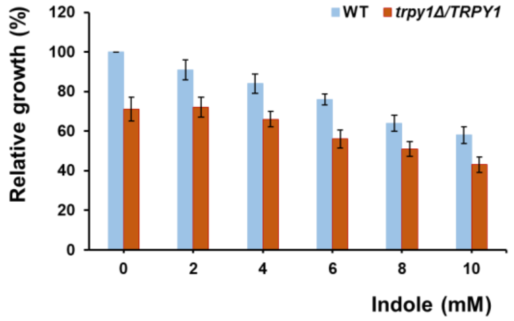

(**j**)

Figure S1. Effect of various substances on the haploinsufficiency of the heterozygous *trpy1Δ*/*TRPY1*. Wild type BY4743 (*TRPY1*/*TRPY1*) and isogenic heterozygous *trpy1Δ*/*TRPY1* diploid strains were shifted to LMeMM (final OD_{600} = 0.05) and grown for 2 hours before chemicals were added to the desired concentration from sterile stocks. Cell growth was recorded spectrophotometrically 24 hours after the addition of the chemicals and normalized to the growth of WT in the absence of chemicals. Effect of: (**a**) Ca^{2+}; (**b**) Cu^{2+}; (**c**) Fe^{3+}; (**d**) Mn^{2+}; (**e**) Zn^{2+}; (**f**) EGTA, Ca^{2+} chelator; (**g**) Gd^{3+}, Ca^{2+} channel blocker; (**h**) Ascorbate (antioxidant); (**i**) Glutathione; (**j**) Indole.
